# Efficacy of GP referral of insufficiently active patients for expert physical activity counseling: protocol for a pragmatic randomized trial (The NewCOACH trial)

**DOI:** 10.1186/s12875-014-0218-1

**Published:** 2014-12-29

**Authors:** Erica L James, Ben Ewald, Natalie Johnson, Wendy Brown, Fiona G Stacey, Patrick Mcelduff, Angela Booth, Fan Yang, Charlotte Hespe, Ronald C Plotnikoff

**Affiliations:** School of Medicine and Public Health, University of Newcastle, Callaghan, NSW Australia; Priority Research Centre for Health Behaviour, University of Newcastle, Callaghan, NSW Australia; Priority Research Centre in Physical Activity and Nutrition, University of Newcastle, Callaghan, NSW Australia; Hunter Medical Research Institute, University of Newcastle, Callaghan, NSW Australia; School of Human Movement Studies, University of Queensland, St Lucia, QLD Australia; Inner West Sydney Medicare Local, Ashfield, NSW Australia; General Practice Research, School of Medicine, Sydney, The University of Notre Dame, Darlinghurst, NSW Australia

**Keywords:** Physical activity, Primary care, Referral, Pedometry

## Abstract

**Background:**

Physical inactivity is fourth in the list of risk factors for global mortality. General practitioners are well placed to offer physical activity counseling but insufficient time is a barrier. Although referral to an exercise specialist is an alternative, in Australia, these allied health professionals are only publicly funded to provide face-to-face counseling to patients who have an existing chronic illness. Accordingly, this trial aims to determine the efficacy of GP referral of insufficiently active patients (regardless of their chronic disease status) for physical activity counseling (either face-to-face or predominately via telephone) by exercise specialists, based on patients’ objectively assessed physical activity levels, compared with usual care. If the trial is efficacious, the equivalence and cost-effectiveness of face-to-face counseling versus telephone counseling will be assessed.

**Methods:**

This three arm pragmatic randomized trial will involve the recruitment of 261 patients from primary care clinics in metropolitan and regional areas of New South Wales, Australia. Insufficiently active (less than 7000 steps/day) consenting adult patients will be randomly assigned to: 1) five face-to-face counseling sessions, 2) one face-to-face counseling session followed by four telephone calls, or 3) a generic mailed physical activity brochure (usual care). The interventions will operationalize social cognitive theory via a behavior change counseling framework. Participants will complete a survey and seven days of pedometry at baseline, and at three and 12 months post-randomization. The primary analyses will be based on intention-to-treat principles and will compare: (i) mean change in average daily step counts between baseline and 12 months for the combined intervention group (Group 1: face-to-face, and Group 2: telephone) and usual care (Group 3); (ii) step counts at 3 months post-randomization. Secondary outcomes include: self-reported physical activity, sedentary behavior, quality of life, and depression.

**Discussion:**

If referral of primary care patients to exercise specialists increases physical activity, this process offers the prospect of systematically and sustainably reaching a large proportion of insufficiently active adults. If shown to be efficacious this trial provides evidence to expand public funding beyond those with a chronic disease and for delivery via telephone as well as face-to-face consultations.

**Trial registration:**

Australian New Zealand Clinical Trials Registry ACTRN12611000884909.

## Background

Physical inactivity is fourth in the list of risk factors for global mortality [1]. In 2011–2012, fewer than 1 in 5 Australian adults met the threshold of 10,000 steps per day to confer a health benefit, with the mean (pedometer-assessed) step count being 7,400 steps per day [2,3]. As most (90%) Australian adults visit a General Practitioner (GP) at least once a year [4], GPs are well placed to provide physical activity counseling. However, evidence regarding the effectiveness of physical activity counseling by medical professionals is mixed [4-6]. Only behavioral counseling interventions involving greater than 30 minutes of total patient contact time have shown beneficial effects on behavioral and intermediate health outcomes, and only interventions with more than 360 minutes of total patient contact time reported sustained benefits beyond 12 months [6]. Effective physical activity counseling by GPs may not be feasible given that GPs generally identify a lack of time as a barrier to preventive counseling [7-14].

One alternative to physical activity counseling by GPs is the referral of patients to an exercise professional (variously referred to as ‘exercise on prescription’, ‘GP exercise referral’ or just an ‘exercise referral’ scheme). Although variations in delivery exist, exercise referral commonly involves a GP (or another member of the primary care team) identifying and referring a sedentary individual with evidence of at least one cardiovascular risk factor or existing chronic disease to a third party service (often a sports centre or leisure facility) [15]. This service then prescribes and monitors an exercise program (normally 12–14 weeks) often delivered at the sports/leisure centre [16]. In the United Kingdom, contemporary exercise referral schemes were first set up around 1990, with more than 600 schemes now in operation [17] all adhering to a core set of standards [18]. The exercise referral model is also being established in primary care practice in other parts of Europe [19,20] and is operationalized differently internationally. In New Zealand, referral schemes have tended to be delivered as part of ‘Green Prescription’ schemes administered through primary care settings with the support of trained telephone counselors [21,22]. The Green Prescription is based around achieving daily time-based activity goals and has been shown to be efficacious in increasing and maintaining physical activity 12 months post-prescription [21] and is cost-effective both in terms of relative cost per quality adjusted life year [23] and per successful treatment [24].

In Australia, the Federal Government has incorporated referral to Exercise Physiologists (EPs) as part of the government-funded Medicare scheme for adults with a diagnosed chronic disease (originally known as an Enhanced Primary Care Plan and now referred to as Chronic Disease Management (CDM) [25]). EPs are university qualified allied health professionals with skills in sports physiology and training and rehabilitation as well as in clinical exercise interventions for persons at high-risk of developing, or with existing chronic and complex, medical conditions and injuries. EPs that undergo additional supervised training and meet the accreditation criteria set by Exercise and Sports Science Australia (ESSA) are eligible to register with Medicare to provide subsidized services as part of CDM. Under CDM, GPs develop a care plan that includes referral to a range of allied health professionals with patients eligible for up to five Medicare-funded sessions with the identified allied health professionals in any calendar year. Patient eligibility for CDM is restricted to those with a chronic medical condition that has been (or is likely to be) present for six months or longer. Despite the availability of this program, only 1% of GP consultations are related to chronic disease management [4], and EP consultations occur at a national rate of 2.6 consultations per 1000 people [26]. There have been few evaluations of the efficacy or cost-effectiveness of the CDM planning and referral pathway [27-29].

A recent systematic review and meta-analysis of eight randomized controlled trials (5190 participants) assessing the impact of exercise referral schemes concluded that considerable uncertainty remains as to the efficacy of referral schemes for increasing physical activity, fitness or health indicators for people with or without a medical diagnosis, and whether they are an efficient use of resources [17]. The review authors concluded there was significant heterogeneity across the current trials and recommended the conduct of further trials of these schemes, particularly ones that incorporate theory-driven interventions [17]. In addition, since all but one of the included 8 trials relied on a self-report measure of physical activity, trials that use an objective measure of physical activity are required. All eight of the trials in the Pavey review [17] referred inactive patients to a structured, supervised exercise program that was typically 10–12 weeks in duration, and held at leisure centres, clinics, or parks. None of the trials utilized individual coaching by a qualified exercise professional, and the development of an individualized unsupervised exercise plan. Only three trials had follow-ups of 12 months, and none longer than 12 months.

Anokye and colleagues [30] examined the cost-effectiveness of exercise referral schemes and concluded that whilst they are associated with a modest increase in lifetime costs and benefits, the cost-effectiveness is highly sensitive to small changes in the effectiveness and cost of schemes. It is therefore important to evaluate the various models of referral and compare cost-effectiveness for different delivery modes (e.g., face-to-face, telephone).

The NewCOACH pragmatic randomized controlled trial described here has two aims. Firstly to determine the efficacy of GP referral of insufficiently active patients (regardless of their chronic disease status) for physical activity counseling by EPs (either face-to-face or predominately via telephone), based on patients’ objectively assessed physical activity levels, compared with usual care. Secondly to compare the equivalence and cost-effectiveness of face-to-face and telephone counseling.

## Methods

### Design

**Figure 1:**
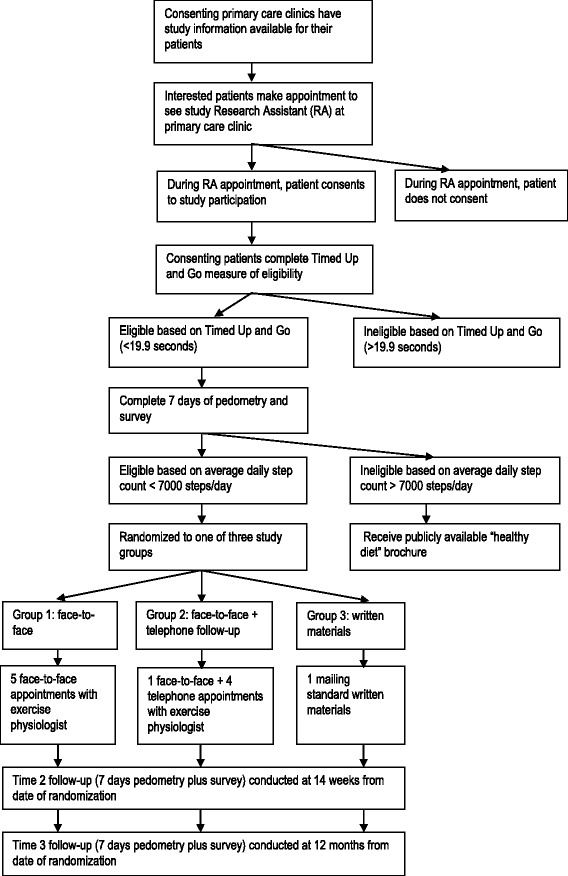
**Flowchart of study participation.**

The study was a three arm, parallel group, individually randomized pragmatic trial designed to determine the effect of physical activity counseling by an EP for patients referred from primary care (see Figure 1). Follow-up assessments occurred three and 12 months post-randomization. A pragmatic trial (a term first coined by Schwartz and Lellouch in 1967) is a randomized controlled trial whose purpose is to inform decisions about practice [31]. The current trial was deemed pragmatic as it was: 1) examining the intervention when used in normal practice, had little restriction on participants (e.g., inclusion of overweight/obese participants or those with multiple comorbidities); 2) it was conducted in the usual primary care setting with no additional resourcing; 3) the intervention (exercise counseling) delivered by EPs was applied flexibly based on their professional judgement and the preferences of the participant, and; 4) the outcomes (physical activity behavior at 12 months, cost) are directly relevant to participants, healthcare providers and funders [31]. This protocol follows recommended reporting procedures [31,32].

### Recruitment

#### Recruitment of primary care clinics

All primary care clinics situated in Newcastle and the surrounding areas of Lake Macquarie, and the lower Hunter (Maitland) were identified via lists held at a regional organization for primary care (Hunter Medicare Local) and via cross-checking with the local white pages electronic phone directory. All practices were invited by telephone to participate. In line with recommendations for conducting research in primary care [33], primary recruitment was conducted by a practicing GP (Chief Investigator Ewald) who had clinical credibility with the target group. CI Ewald attended a practice meeting at interested clinics to describe the aims of the study and the requirements of participating practices. A second recruitment pool in Inner West Sydney was established through partnership with the Inner West Sydney Medicare Local. The Inner West Sydney Medicare Local held lists of all clinics in the local area, and all GPs were invited to attend a dinner to hear CI Ewald describe the study. For GPs that expressed interest in the research study but were unable to attend the GP dinner, a separate face-to-face meeting was scheduled to provide information about the research study. Participating GP clinics in both areas were offered a small fee (AUD$50 for each day) to cover administration expenses for the use of an available room for the research assistant to meet with potential participants.

#### Recruitment of participants

Patients attending appointments at participating practices were advised about the study by GPs, practice nurses, receptionists, and via promotional materials placed in the waiting room and reception area, or a combination of these strategies. The aim was for GPs to identify potentially eligible patients and initiate discussion of the study with them. After discussion with their GP, interested patients were asked to book an appointment on one of the pre-determined recruitment days to speak to a research assistant about the study. There has been a blurring in previous trials between ‘GP referral’ and ‘recruitment from the primary care setting’. The first maximizes the influence of the doctor-patient relationship and inherently involves the clinician in patient care, whilst the second utilizes the primary care setting to identify potentially eligible patients, and may or may not involve the treating clinician (e.g., having a research assistant recruit patients from the clinic waiting room). To maintain the method most closely resembling real world practice and to ensure a genuine GP referral pathway, situating a research assistant in the waiting room for recruitment was not employed in the current trial. This pragmatic approach needed to be balanced with limiting the amount of time required by GPs or other practice staff to undertake patient screening (that related to the research trial and would not be necessary in usual practice for a referral scheme) [33]. For this reason, following identification and referral of the potentially eligible patient, a study-specific research assistant undertook all study screening and consent-related activities during a scheduled appointment at the practice rooms.

### Eligibility and screening

During the scheduled appointment, the research assistant explained the study and obtained informed consent from patients who met the following eligibility criteria: 18 years of age or older, with regular access to a telephone, able to complete the study measures and interventions in English, plan to live within the defined geographic recruitment area for the following 12 months and willing to undergo screening. Patients needed to be able to safely participate in either of the interventions (face-to-face only, or face-to-face and telephone).

A two-stage screening process was used. Firstly, during the scheduled appointment with the research assistant, consenting patients were screened using the Timed Up and Go three metre test [34]. Patients who took longer than 19.9 seconds [34] to rise from a chair, walk three metres, turn around, and walk back were excluded as they were considered too frail or disabled to begin an unsupervised physical activity program without appropriate rehabilitation. The second stage of screening occurred after the appointment and required the patient to wear a pedometer and record on a log sheet their step count for seven days. Patients posted back the completed log sheet and, after assessment by the research manager, were excluded if they achieved more than an average of 7000 steps per day [35]. This was a more stringent eligibility measurement of current PA than the commonly utilized self-reported method of screening but was deemed necessary to: a) ensure sample were insufficiently active at baseline and therefore avoid potential ceiling effect; and b) exclude potential participants who were very active and may have been interested in accessing specialist exercise advice to enhance performance.

### Randomization and blinding

Random allocation of eligible participants was conducted in two stages, and in order to offer participants a service that was geographically accessible they were asked to indicate the suburbs where they would prefer to see an EP. First, eligible patients were randomly assigned in a 1:2 ratio to the usual care group or to the intervention group using a randomized block design stratified by participant gender with block sizes of 6. Second, those allocated to the intervention group were randomly assigned in a 1:1 ratio to face-to-face only or face-to-face and telephone counseling using a randomized block design stratified by participant gender and the suburb location of the EP, using block sizes of 6. The randomization process occurred on computer at a centralized location by an independent statistician. Participants were notified of group allocation by letter. Central randomization allowed concealment of allocation from research assistants and GPs until group assignment had occurred. Blinding of participants and EPs was not feasible. Contamination was minimized as the intervention was not delivered at the primary care site and follow-up data collection occurred by mail, with patient-reported survey and pedometer-assessed step counts, so was not influenced by study team members. The data analyst was blinded to group allocation, as surveys were de-identified and did not contain study group information.

### Interventions

Physical activity behavior change counseling was guided by Bandura’s Social Cognitive Theory (SCT) [36]. The constructs operationalized as part of the intervention included: self-efficacy, outcome expectations, social support, perceived physical environment, attitudes, confidence to change behavior, goals and intentions.

The physical activity behavior change counseling was delivered by accredited EPs in private practice. All accredited EPs working within the geographic areas were invited to participate in the study. EPs provided the following information: a curriculum vitae, two referees, current professional indemnity and public liability insurance certification, and proof of professional membership of ESSA. EPs attended a five hour study-specific training session conducted by members of the research team with expertise in theory-driven behavior change and communication skills. The training comprised a mix of oral presentations, written information, role plays, feedback, and small group discussion, based on effective evidence-based physician training strategies [37]. The content of the training session covered the importance of behavior change theory (especially SCT) and how to operationalize SCT constructs using a patient-centred behavior change counseling approach.

#### Group 1: referral for face-to-face physical activity counseling

Participants randomized to Group 1 attended five face-to-face consultations with an EP over the 13-week intervention period (13 weeks from date of randomization). The initial consultation was approximately 60 minutes, and each follow-up appointment was approximately 30 minutes in duration. The EP consultations were at no cost to participants, and EPs were reimbursed by the research team at a rate of AUD$90 per initial consultation and AUD$55 per follow-up consultation. Consultations occurred at the EPs private practice. The initial consultation comprised an assessment of the participant’s ability to safely undertake physical activity, and the application of behavior change strategies to enhance participation in physical activity, such as goal setting, the identification of barriers to physical activity and collaborative problem-solving to identify strategies to overcome personal barriers to physical activity. At follow-up visits, progress against goals was assessed, new goals set, and any challenges discussed using the same collaborative problem-solving approach. All consultations were patient-centred and tailored to the participant’s physical activity preferences, capability, medical limitations (if present) and personal barriers. Discussion of cognitive and behavioral strategies, derived from SCT, occurred as appropriate to each person’s motivational readiness to change. Beyond the training in patient-centred counseling and behavior change strategies, there was no other attempt to standardize the intervention and its delivery across participants, EPs, or geographic location.

#### Group 2: referral for face-to-face and telephone physical activity counseling

Participants randomized to Group 2 attended one face-to-face consultation (60 minutes) with an EP and participated in four telephone counseling sessions with the same EP over the 13-week intervention period. The telephone calls were expected to take 30 minutes. The aims and content of the initial and subsequent consultations were the same as for Group 1.

If an EP assessed during the initial face-to-face consultation that the patient in either Group 1 or Group 2 had a complex medical issue that would prevent them from being able to safely undertake increased exercise without appropriate supervision, then participants were deemed ineligible, and were excluded from the intervention and follow-ups.

#### Group 3: usual care

Group 3 received usual care from their GP and were mailed a generic health promotion brochure promoting physical activity and outlining the National Physical Activity Guidelines for Australians [38].

### Intervention fidelity and process evaluation

During each EP consultation, the EP completed a checklist of topics covered during the consultation. Consultation checklists were returned to researchers with invoices for each of the consultations. The checklists were used to assess: the number of sessions completed with each participant, and the duration of each consultation, and any falls or injuries. The checklists also provided information on whether any of the following SCT strategies were discussed during each consultation: confidence to change behavior, goal setting, physical activity planning, goal review and monitoring, overcoming barriers, sources of social support, and sources of environmental support.

### Outcomes

Participants completed seven days of pedometry and completed a pen and paper survey at three and 12 months post-randomization.

### Primary outcome

The primary outcome was change in step counts, as measured by seven days of pedometry at baseline and 12 months post-randomization. Participants were instructed to wear an unsealed G-Sensor 2025 pedometer during waking hours and to record the total daily steps in a log. Pedometers have been shown to be a cost-effective option for objectively assessing activity by measuring step counts [39]. Daily step counts were recorded on a log sheet, along with duration of other physical activity that a pedometer does not capture, such as swimming or cycling. These other activities were converted via their respective MET values to equivalent step counts [40], and included in the calculation of average daily step counts. To increase the accuracy of activities not captured by pedometer, participants were asked to report the number of laps swum (50 metre pool), and the number of kilometres cycled. Each lap swum was deemed equivalent to 363 steps and each kilometre cycled was deemed equivalent to 878 steps. All ‘other’ activities were coded using previously reported step count values (table two) [41]. Average daily step count comprised the total step count, including ‘other activities’, divided by the number of days with 10 or more hours of wear time. Every pedometer was tested for accuracy before every use, on a calibration machine, and had to measure 100 cycles of simulated steps within plus or minus two cycles. Whilst there is concern over the reactivity of participants to wearing pedometers, i.e. walking more during the time they are wearing the device [42], any reactivity should be the same at baseline and follow up. At recruitment, participants could choose to be sent an SMS each morning reminding them to wear the pedometer.

### Accelerometer sub-study

A sub-sample of participants was asked to wear an accelerometer, in addition to the pedometer. Consenting participants were provided with a three axis GeneActiv accelerometer worn at the wrist, recording at 40 Hz at each time point in addition to other study measures. Accelerometer data will be used to describe sitting time, and time spent at different activity intensities, and to validate self-reported steps and PA.

### Demographic and secondary outcomes

#### Self-reported physical activity

Physical activity frequency and duration were measured using eight items from the Active Australia survey [43]. The survey evaluates four types of physical activity in the last week: walking, moderate physical activity, vigorous physical activity, and vigorous gardening or heavy work [43]. The Active Australia survey has demonstrated reliability and validity and acceptable test-retest reliability [44].

#### Sedentary behavior

Sedentary behavior was measured using five items asking about time spent sitting (hours and minutes) during the last working and non-working day in each of the following domains: (a) while traveling to and from places; (b) while at work; (c) while watching television; (d) while using a computer at home; and (e) at leisure not including watching television (e.g., visiting friends, movies, eating out) [45]. The five item measure has demonstrated reliability and validity for sitting time on weekdays, but is less reliable for weekends and less structured leisure activities [45].

#### Quality of life

Quality of life was assessed using the AQoL 8D 35-item survey, which measures quality of life across eight dimensions: independent living, relationships, mental health, coping, pain, sense, life satisfaction, and self-worth [46]. The AQoL 8D has been shown to have strong validity [47] and good reliability [48].

#### Depression

The Centre for Epidemiologic Studies Depression Scale (CES-D) 20-item scale was used to measure depressive symptoms. It was designed to measure depressive symptoms in the general population, and it has acceptable reliability and validity [49].

#### Potential mediators of physical activity

A three month time reference was provided for all of the potential mediators (based on SCT) of physical activity. For purposes of mediation analysis, “regular physical activity” was defined as “30 minutes or more per day over at least 5 days per week, and adds up to 150 minutes or more per week” which is consistent with national guidelines for Australian adults [38].

- Goals and intentions – Behavioral goal was measured with one item which asked “How likely is it that you will do ‘regular physical activity’ within the next 3 months?” on a scale from 0-100% [50]. To assess action planning, four items were designed to measure plans made in relation to regular physical activity, with a five point Likert-type scale from 1 ‘Not at all’ to 5 ‘Very much’. These four questions have internal reliability ranging from 0.92 to 0.95 [51].
- Self-efficacy – Nine items were used to measure confidence to participate in regular physical activity, and were measured on a five point Likert-type scale from 1 ‘Not at all’ to 5 ‘Extremely’ [52]. The items show internal reliability from 0.88 to 0.90 [52].
- Outcome expectations – Ten items were used to measure outcome expectations, on a five point Likert scale from 1 ‘ Strongly disagree’ to 5 ‘Strongly agree’. Five measured positive outcome expectations and five measured negative outcome expectations [52]. With Cronbach alphas of 0.79 for the positive scale, and 0.71 for the negative scale, these measures have adequate validity and reliability for use in a general adult population [52].
- Social support – Two items were used to measure social support in relation to participating in regular physical activity on a five point Likert-type scale from 1 ‘Not at all’ to 5 ‘Very much’ [50] with a correlation of 0.57 in the total sample [53].
- Perceived environmental local surroundings – Nine items were used to assess the local suburb’s physical surroundings (1. Greenery; 2. Interesting things to look at; 3. Pleasant natural features; 4. Footpaths on most streets; 5. Footpaths that are well-lit at night; 6. Lots of traffic; 7. Live near busy road; 8. Unsecured dogs; 9. Feel safe walking home at night) [54]. The reliability of these nine items has been reported as moderate to substantial, with individual item ICCs ranging from 0.51 to 0.74 [54].

### Socio-demographic and medical characteristics

Thirty questions were used to assess socio-demographic and medical characteristics (date of birth, country of birth, gender, marital status, level of education, income, work status), waist circumference (self-measured using a provided paper tape measure and standardized instructions), weight, height, health status and lifestyle behaviors, dog ownership and time spent walking with the dog, and participant preferences for study group allocation. Where possible, survey items were taken directly from, or based on, validated surveys [55-58].

### Contamination and adverse events

At three month and 12 month follow-up surveys, participants were asked to report on their use of any physical activity health education or promotion programs, the factors that influenced their physical activity levels since the previous survey, and recall any falls or injuries since the previous survey. The checklist completed by EPs after each consultation also prompted recording of any falls or injuries suffered by the participant.

### Follow-up and minimization of attrition

Since attrition reduces the effective sample size and can introduce bias, strategies shown to increase response to questionnaires in a Cochrane review were employed [59]. Accordingly, if the completed survey, pedometer and pedometer log sheet was not received after two weeks, participants received a reminder postcard. If not received after a further two weeks, participants received a reminder telephone call or reminder email, and a second copy of the questionnaire was mailed, if requested. Participant attrition and adverse events were monitored at each stage of the trial by the Research Manager.

### Sample size

A sample size of 79 participants per group will provide 90% power to detect a differential increase of 1000 steps per day at 12 months between the two intervention groups and the usual care group at the 5% significance level. This sample size was calculated assuming that the usual care group will increase their average daily step count by 1000 steps, and the standard deviation of 2500 observed in the pilot study. Attrition rates are estimated to be 10% over the twelve month period [60]. This results in a total required sample of 261 (n = 87 per group).

### Statistical analyses

Baseline data will be summarized as the number of observations, means, standard deviations, medians, minimums and maximums where the data are continuous and as number of observations and frequencies where the data are categorical. The data will be presented separately by treatment group.

The primary endpoint (mean change in daily step counts from baseline to 12 months) will be analyzed using a constrained linear mixed model approach [61], where the outcome in the model is the mean number of steps per day at each time point, but the baseline mean responses for the treatment groups are assumed equal. This approach has been shown to be robust when missing data depend on baseline values [62]. Predictor variables in the model will include fixed effects for group (usual care as reference and group 1: face-to-face, group 2: telephone), time (baseline as reference, 3 months and 12 months) and the interaction between group and time. A subject level random intercept will be included in the model to allow for correlations arising from repeated measures. There may be some variation to this model after checking its underlying assumptions of multivariate normality, linearity and equal variance and clustering. The primary comparison will be between the combined intervention groups (group 1: face-to-face and group 2: telephone) and usual care. A secondary analysis will examine the difference in step counts at 3 months, and if significant differences are found in the primary analysis, will also explore if there are differences between treatment groups (face-to-face and telephone). Sub group analyses will be conducted by sex, BMI category and diagnosed chronic disease versus none. Sensitivity analyses will explore the impact of individual EP, GP clinic, and after removing the steps imputed from self-reported physical activity. The analysis will be conducted on an intention-to-treat basis with alternative methods of accounting for missing data: multiple imputation, and pattern mixture modelling [63].

Process evaluation will be assessed by calculating adherence with intervention (mean number of sessions completed, average time of intervention delivery for face-to-face and telephone counseling sessions). Intervention fidelity will be assessed by coding of EP checklists.

### Economic analysis

Economic analysis from the health funder perspective will form part of this trial. Costs of the intervention will be derived from the time spent by the EPs on consultations in person and by phone, and on record keeping, and will include the time spent by the GP making the referral. Utility will be derived from the AQoL responses transformed into Quality Adjusted Life Years (QALYs) and the incremental cost per QALY will be calculated.

### Secondary analyses

i. An exploration of whether change in physical activity behavior results in spontaneous change in other health behaviors (smoking, alcohol consumption).
ii. An examination of the predictors of participants who adhere with the NewCOACH intervention.
iii. Mediation and moderation analyses to identify factors associated with physical activity behavior change.
iv. An exploration of whether there is any difference in intervention efficacy between people who receive their preferred study group allocation and those who do not.
v. The impact of the physical activity intervention on quality of life, depression, and fatigue.
vi. Relationships between changes in step count and changes in accelerometer-derived sitting time.

### Ethics

The study has been approved by the University of Newcastle’s Human Research Ethics Committee (H-2011-0063).

## Discussion

The NewCOACH study is a three-arm pragmatic randomized trial that aims to determine the efficacy of GP referral of insufficiently active patients (regardless of their chronic disease status) for physical activity counseling (either face-to-face or predominately via telephone) by exercise specialists, based on patients’ objectively assessed physical activity levels, compared with usual care. If the trial is efficacious, the equivalence and cost-effectiveness of face-to-face counseling versus telephone counseling will be assessed.

Strengths of this study include the pragmatic design, the use of an objective measure of physical activity, the use of qualified exercise specialists (EPs) to deliver the theory-based behavior change counseling, and the 12 month follow-up to assess maintenance of behavior change. Limitations of the study include that only a sub-sample will provide accelerometer data.

If the physical activity counseling is efficacious, this will have implications for the design of future exercise referral schemes and EP funding models both in Australia and internationally. If the trial demonstrates that referral to an EP for coaching is an effective way to get people more active, it will be very attractive to GPs to make greater use of CDM pathways. Current funding legislation in Australia restricts CDM to those with an existing chronic disease [64]. If improvements in physical activity can be demonstrated for previously inactive but otherwise healthy adults in this trial, this will provide support for expansion in the eligibility criteria for the Medicare allied health initiative to include insufficiently active patients who do not have a chronic condition. This would be a significant shift away from just treatment of disease, and would acknowledge the role of allied health professionals in preventing disease in partnership with primary care clinicians. In addition, Medicare funding currently only recognizes face-to-face consultations. Evidence that telephone coaching is equally or more effective could lead to the funding of EP services for telephone delivery which would increase the reach and flexibility of this service.

## Abbreviations

GPs: General practitioners
CDM: Chronic disease management
EP: Exercise physiologist
ESSA: Exercise and Sports Science Australia
SCT: Social cognitive theory
ITT: Intention to treat
QALY: Quality adjusted life years
